# Bromodomain-Containing Protein 9 Regulates Signaling Pathways and Reprograms the Epigenome in Immortalized Human Uterine Fibroid Cells

**DOI:** 10.3390/ijms25020905

**Published:** 2024-01-11

**Authors:** Qiwei Yang, Somayeh Vafaei, Ali Falahati, Azad Khosh, Maria Victoria Bariani, Mervat M. Omran, Tao Bai, Hiba Siblini, Mohamed Ali, Chuan He, Thomas G. Boyer, Ayman Al-Hendy

**Affiliations:** 1 Department of Obstetrics and Gynecology, University of Chicago, 5841 S. Maryland Ave., Chicago, IL 60637, USA; somayehv@bsd.uchicago.edu (S.V.); bariani@bsd.uchicago.edu (M.V.B.); mervatomran@bsd.uchicago.edu (M.M.O.); hsiblini@bsd.uchicago.edu (H.S.); mohamed.ali@bsd.uchicago.edu (M.A.); aalhendy@bsd.uchicago.edu (A.A.-H.); 2 DNA GTx LAB, Dubai Healthcare City, Dubai 505262, United Arab Emirates; a.falahati@dnagtx.com; 3 Department of Molecular Medicine, Institute of Biotechnology, University of Texas Health Science Center at San Antonio, San Antonio, TX 78229, USA; khosh@livemail.uthscsa.edu (A.K.); boyer@uthscsa.edu (T.G.B.); 4 Cancer Biology Department, National Cancer Institute, Cairo University, Cairo 11796, Egypt; 5 Obstetrics and Gynecology, Feinberg School of Medicine, Northwestern University, Chicago, IL 60611, USA; tao.bai@northwestern.edu; 6 Department of Chemistry, University of Chicago, Chicago, IL 60637, USA; chuanhe@uchicago.edu

**Keywords:** uterine leiomyoma, BRD9, I-BRD9, cell proliferation, apoptosis, extracellular matrix, transcriptome, epigenome, chromatin remodeling, m^6^A regulators, epitranscriptome, RNA methylation

## Abstract

Bromodomain-containing proteins (BRDs) are involved in many biological processes, most notably epigenetic regulation of transcription, and BRD dysfunction has been linked to many diseases, including tumorigenesis. However, the role of BRDs in the pathogenesis of uterine fibroids (UFs) is entirely unknown. The present study aimed to determine the expression pattern of BRD9 in UFs and matched myometrium and further assess the impact of a BRD9 inhibitor on UF phenotype and epigenetic/epitranscriptomic changes. Our studies demonstrated that the levels of BRD9 were significantly upregulated in UFs compared to matched myometrium, suggesting that the aberrant BRD expression may contribute to the pathogenesis of UFs. We then evaluated the potential roles of BRD9 using its specific inhibitor, I-BRD9. Targeted inhibition of BRD9 suppressed UF tumorigenesis with increased apoptosis and cell cycle arrest, decreased cell proliferation, and extracellular matrix deposition in UF cells. The latter is the key hallmark of UFs. Unbiased transcriptomic profiling coupled with downstream bioinformatics analysis further and extensively demonstrated that targeted inhibition of BRD9 impacted the cell cycle- and ECM-related biological pathways and reprogrammed the UF cell epigenome and epitranscriptome in UFs. Taken together, our studies support the critical role of BRD9 in UF cells and the strong interconnection between BRD9 and other pathways controlling the UF progression. Targeted inhibition of BRDs might provide a non-hormonal treatment option for this most common benign tumor in women of reproductive age.

## 1. Introduction

Uterine fibroids (UFs) are the most common benign tumors in women of reproductive age. UFs are heterogeneous in composition, size, and number among individual women and within the same individual. UFs occur in ~77% of women overall and are clinically manifest in ~25% by age 45 [1,2,3]. Although benign, these tumors are nonetheless associated with significant morbidity; they are the primary indicator of hysterectomy and a major source of gynecologic and reproductive dysfunction, ranging from menorrhagia and pelvic pain to infertility, recurrent miscarriage, and pre-term labor [4]. Accordingly, the annual US healthcare costs associated with UFs have been estimated at ~$34 billion. UFs thus represent a significant societal health and financial burden.

Epigenetic dysregulation is often linked to human disease, notably tumorigenesis, which is the consequence of the combined action of multiple epigenetic events [5]. Accumulated evidence has demonstrated that dysregulation of epigenetic modifications, including alterations to DNA methylation, non-coding RNAs, and histone modifications, play a critical role in the pathogenesis of UFs [6,7,8,9,10,11,12,13,14]. Modifications to histones, including methylation, phosphorylation, ubiquitination, and acetylation, can directly influence chromatin structure. For instance, lysine acetylation neutralizes the positive charge of histones, therefore repressing their interaction with negatively charged DNA and reinforcing nucleosome fluidity [15]. Notably, bromodomain -containing proteins (BRDs) primarily recognize acetylated lysine on histones and regulate gene expression via multiple gene regulatory mechanisms [9,16,17,18,19,20,21,22]. So far, researchers have identified 46 BRDs with a total of 61 bromodomains in the human proteome. Human BRD modules are classified into eight families, based on their structural topology and sequence similarity [23,24]. For instance, the bromodomain and extra terminal (BET) family in subgroup II play an important role in many biological events and the development of diseases. The four members of the BET protein family, including BRD2, BRD3, BRD4, and BRDT, share a common domain consisting of two N-terminal bromodomains, which bind to acetylated lysine residues on histones [25,26,27]. Recent studies have demonstrated that non-BET proteins are important in diverse human diseases, including cardiovascular disease, inflammation, and tumorigenesis [28].

Mammalian SWI/SNF (switch sucrose non-fermentable) (mSWI/SNF) is a family of ATP-dependent chromatin remodeling complexes, which regulates chromatin architecture to enable DNA accessibility, ensuring timely and appropriate control of gene expression. The non-BET BRD9, containing a single bromodomain, is one of the key subunits of the mSWI/SNF ATP-dependent chromatin remodeling complexes involved in coordinating spatial and temporal control of multifaceted protein–protein interactions for chromatin remodeling [29,30]. Notably, BRD9 has been reported to play an important role in cell differentiation [31,32], facilitate the progression of multiple malignancies [33,34,35], and play a significant role in other diseases, such as inflammation [30]. Recently, BRD9 has been shown to be associated with the pathogenesis of uterine leiomyosarcoma, suggesting that BRD9 may have an impact on uterine disorders [36].

Epigenetic-targeted therapy has been applied in treating various diseases, including tumorigenesis. Therefore, developing and using small chemical inhibitors are fundamental and critical to the preclinical evaluation of BRDs as targets. Recently, significant success has been achieved in designing and identifying BRD9 inhibitors with benefits impacting [23,37] multiple types of cancer [38,39,40,41]. However, the role and mechanism of BRD9 in the pathogenesis of benign UFs are entirely unknown. Accordingly, the present study aimed to determine whether and how BRD9 protein contributes to abnormal UF growth, with important implications for developing novel treatment options for this most common type of reproductive tumor.

## 2. Results

### 2.1. The Level of BRD9 Protein Is Aberrantly Upregulated in Uterine Fibroids

**Figure 1:**
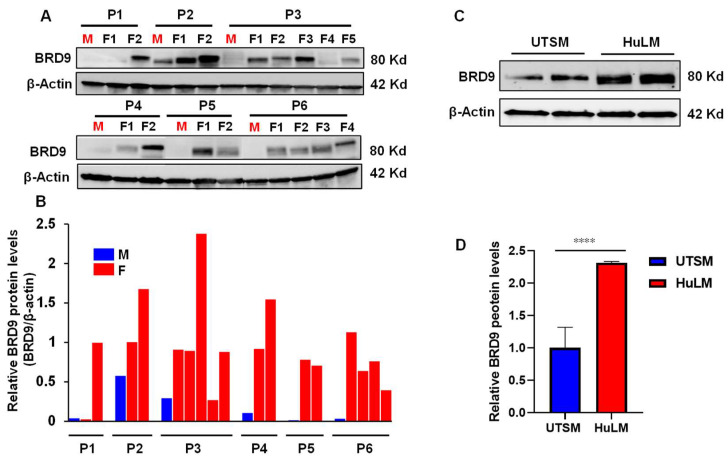
Protein levels of BRD9 in human UF tissues and cells. (**A**) Immunoblot analysis was performed to determine the levels of BRD9 protein in UFs (*n* = 17) and myometrium tissues (*n* = 6). (**B**) The protein levels of BRD9 in UFs and myometrium in (**A**) were quantified using NIH Image J software (1.53t version) (NIH, Bethesda, MD, USA) and presented as fold changes (F/M). (**C**) Immunoblot analysis was performed to determine the levels of BRD9 protein in HuLM and UTSM cells. (**D**) The BRD9 levels in HuLM and UTSM cells in (**C**) were quantified using NIH Image J software and presented as fold changes (HuLM/UTSM). β-actin was used as an endogenous control. P: patients, M: myometrium; F: uterine fibroids, **** *p* < 0.0001.

We first measured the levels of BRD9 protein in human UFs (*n* = 17, UFs from 6 patients) and matched myometrial tissues (*n* = 6). The uterus from each case (cases 1–6) contains multiple UFs. As shown in Figure 1A,B, among 17 UFs analyzed, 81% (15/17, *p* < 0.01) exhibited the upregulation of BRD9 compared to matched myometrium. The BRD9 levels showed differences between UFs from the same uterus. For example, the expression levels of BRD9 in UF#2 are much higher than those of UF#1 for case 1. Similar findings can be observed for other cases, suggesting a heterogeneous characteristic of UFs regarding BRD9 expression. In addition, the expression of BRD9 showed a marked upregulation in the UF cell line (HuLM) compared to the myometrial cell line (UTSM) (Figure 1C,D), suggesting the aberrant BRD protein expression may contribute to the pathogenesis of UFs (*p* < 0.0001).

### 2.2. Inhibition of BRD9 Showed Decreased Cell Proliferation and Anti-Fibrotic Characteristics in Uterine Fibroid Cells

**Figure 2:**
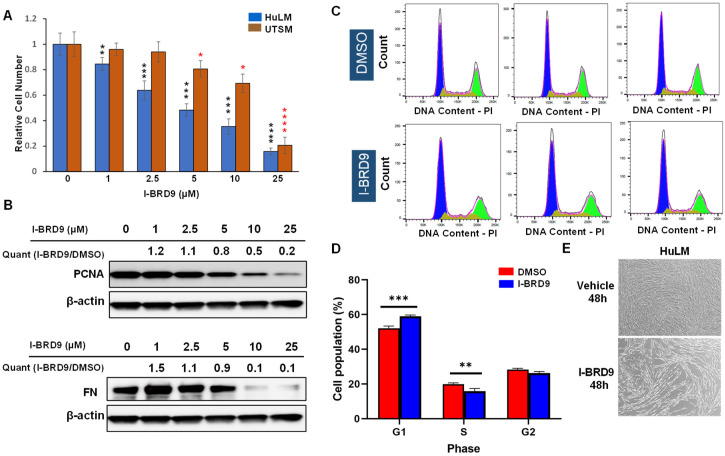
Treatments with I-BRD9 decrease UF cell proliferation and ECM levels. (**A**) HuLM and UTSM cell proliferation was performed in the presence or absence of I-BRD9 with a trypan blue exclusion assay. (**B**) The protein levels of PCNA and fibronectin (FN) were examined via Immunoblot analysis using anti-PCNA and anti-FN antibodies, respectively. Quantification of immunoblot signals was performed after normalization to β-actin. (**C**) Flow cytometry analysis was performed to measure the cell cycle phase distribution (blue color: G1; yellow color: S; green color: G2) in HuLM cells treated with I-BRD9 (*n* = 3 for each group). (**D**) Quantitative analysis of cell cycle data. (**E**) Morphological changes to HuLM cells after treatment with I-BRD9. Magnification was applied ×10. Pictures were taken by EVOS XL Core imaging system (Invitrogen) * *p* < 0.05, ** *p* < 0.01; *** *p* < 0.001; **** *p* < 0.0001. * Comparison between DMSO- and I-BRD9 treated HuLM cells, * comparison between DMSO- and I-BRD9 treated UTSM cells.

To determine the anti-proliferative effect of BRD9 inhibitors on UF cell proliferation, we selected I-BRD9 (the potent BRD9 inhibitor) in our in vitro cell model to assess its impact on UF cell growth. To determine if I-BRD9 affects UF cells and myometrial cells, a trypan blue exclusion assay was performed in HuLM and UTSM cell lines treated with dose ranges from 1–25 µM. Treatment with BRD9 inhibitor (I-BRD9) for 48 h showed a dose-dependent inhibitory effect on the proliferation of UF and myometrial cells (Figure 2A). Notably, I-BRD9 inhibited HuLM cell proliferation in a 1–25 µM dose range. In contrast, I-BRD9 inhibited UTSM cell proliferation in a dose range of 5–25 µM. Moreover, at the same concentration of I-BRD9, a more inhibitory effect can be observed in HuLM cells than in UTSM cells. Overall, UF cells showed a more inhibitory effect on cell proliferation than myometrial cells (Figure 2A). In addition, we measured PCNA levels in response to I-BRD9 treatment within a concentration range of 1–25 µM in HuLM cells. As shown in Figure 2B (up panel), I-BRD9 treatment decreased PCNA levels in a dose-dependent manner, further demonstrating the antiproliferative effect of BRD9 inhibition. Next, to determine if the BRD9 inhibitor exerts an anti-fibrotic effect via decreasing extracellular matrix (ECM) protein levels, we measured fibronectin (FN) levels in the presence or absence of I-BRD9. Compared to vehicle control, I-BRD9 induced a dose-dependent decrease in FN protein levels (Figure 2B, lower panel).

### 2.3. Inhibition of BRD9 Induced Apoptosis, Necrosis, and Cell Cycle Arrest in Uterine Fibroid Cells

I-BRD9 treatment resulted in the increased accumulation of cells in the G1 phase and a corresponding decrease in the S phase, indicating the blockade of G1 progression (Figure 2C,D). The percentage of cells in the G1 phase was increased significantly from 51.5% to 59.0% in response to 5 µM I-BRD9 treatment. Accordingly, the percentage of cells in the S phase significantly decreased from 19.9% to 15.8% in response to I-BRD9 treatment. These results are consistent with the observation that I-BRD9 suppressed cell proliferation, concomitantly with a decrease in the levels of PCNA in HuLM cells.

Moreover, we evaluated the effect of BRD9 inhibitors on apoptosis and necrosis in HuLM cells. As shown in Figure S2A,B, the percentage of cells undergoing early apoptotic cell death increased from 0.9% in the control cells to 1.4% and 1.7% in HuLM cells following 24 h treatment with 1 and 5 µM I-BRD9, respectively. In addition, I-BRD9 exhibited an increase in late apoptosis from 3.7% in the control cells to 6% and 7% in 1 and 5 µM of I-BRD9 treatment, respectively. In addition, the percentage of cells undergoing necrosis increased from 2% in the control cells to 2.5% and 7.1% in HuLM cells after 24 h treatment with 1 and 5 µM of I-BRD9 treatments, respectively (Figure S2).

We also observed that HuLM cells showed elongated morphological changes in response to I-BRD9 treatment compared to the control cells. More space between cells was exhibited in a HuLM cell-containing dish treated with I-BRD9 compared to the control one (Figure 2E).

### 2.4. BRD9 Inhibition Causes Extensive Changes in the UF Cell Transcriptome

#### 2.4.1. Differentially Expressed Genes upon I-BRD9 Treatment

**Figure 3:**
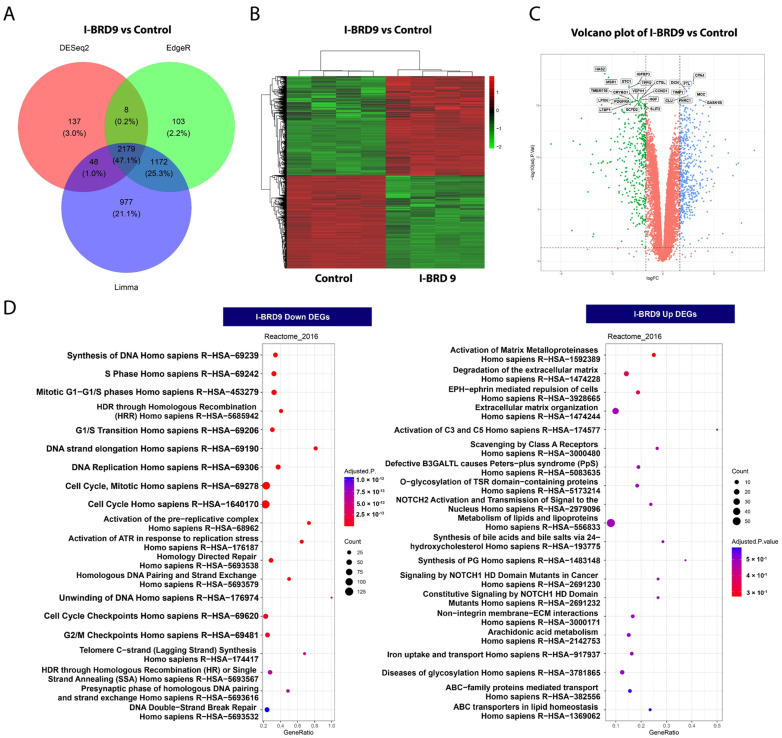
Treatment with I-BRD9 sculpts the transcriptome of UF cells. (**A**) Venn diagrams demonstrating the overlap of DEGs identified via three methods of Limma + voom, edgeR, and DESeq2 at Adjusted *p*-value cut off 0.05 and −1.5 > log2FC > 1.5 for I-BRD9 vs. control. (**B**) Heatmap of I-BRD9 vs. control (DMSO) group. (**C**) Volcano plots of the gene expression profiles of I-BRD9 vs. control. (**D**) Reactome pathway analysis of DEGs. The dot plots show the top twenty enrichment terms associated with down DEGs (**left** panel) and up DEGs (**right** panel) in response to the I-BRD9 treatment. The *x*-axis represents the gene ratio, and the *y*-axis describes the enrichment components. The area of the cycle is proportional to the number of genes assigned to the term, and the color accords with the adjusted *p*-value.

To further investigate the mechanistic basis for the inhibitory action of I-BRD9 in UF cells, RNA sequencing analysis was performed in control (*n* = 4) and I-BRD9 (*n* = 4) treated HuLM cells. Three different programs, namely DESeq2, EdgeR, and limma, were used to characterize the differentially expressed genes (DEGs) for the RNA-seq data set. As there are marked differences between the different algorithms, we obtained three sets of DEGs with considerable variability. In this regard, we used the intersection of three sets representing a conservative estimate of DEGs for downstream analysis. As shown in Figure 3A, the I-BRD9 treatment yielded 2179 DEGs (1056 down, 1123 up). Heatmap analysis demonstrated a distinct expression pattern in HuLM cells treated with I-BRD9 (Figure 3B) compared to the control group. Volcano plot analysis revealed the distribution of changes in response to I-BRD9 (Figure 3C). 

#### 2.4.2. Enrichment Pathway Analysis

We then investigated the DEGs in the control and I-BRD9 groups. Reactome pathway analysis revealed that genes downregulated via I-BRD9 treatment were enriched for pathways, including DNA synthesis/replication and cell cycle progression (S phase, G1/S transition, cell cycle checkpoints) (Figure 3D, left panel). The analysis also demonstrated that genes commonly upregulated via I-BRD9 treatment were enriched for hallmark pathways, including activation of matrix metalloproteinases, degradation of the extracellular matrix, and extracellular matrix organization, which are the hallmarks for UF disease (Figure 3D, right panel). 

**Figure 4:**
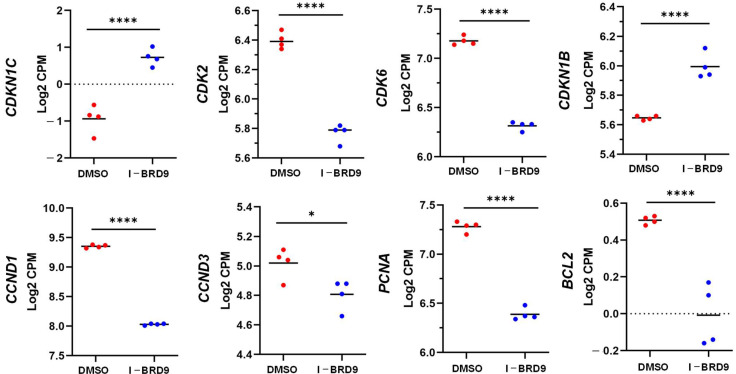
I-BRD9 altered cell cycle- and apoptosis-related gene expression in HuLM cells. RNA-seq revealed the downregulation of *CCND1*, *CCND3*, *CDK2*, *CDK6*, *PCNA,* and *BCL-2*, and upregulation of *CDKN1C* and *CDKN1B*. * *p* < 0.05; **** *p* < 0.0001.

To further determine the molecular mechanism underlying I-BRD9-induced cell cycle arrest, we compared the expression of cell cycle- and apoptosis-related genes between control and I-BRD9-treated HuLM cells. RNA-seq analysis demonstrated that cell cycle- and apoptosis-related gene expression levels, including *CCND1*, *CDK2*, *CDK3*, *CCND3*, *CCND6*, *PCNA*, and *BCL2,* were downregulated in response to I-BRD9 treatment (Figure 4). In contrast, the expression levels of genes encoding cyclin-dependent kinase inhibitors, including *CDKN1B* and *CDKN1C*, were significantly upregulated via BRD9 inhibition (Figure 4). We validated the RNA expression of several cell cycle-related genes, including *CDK2*, *CCND1*, *CCND3*, and *PCNA,* via real-time PCR. The results were consistent with the RNA-seq data (Figure S3A). 

**Figure 5:**
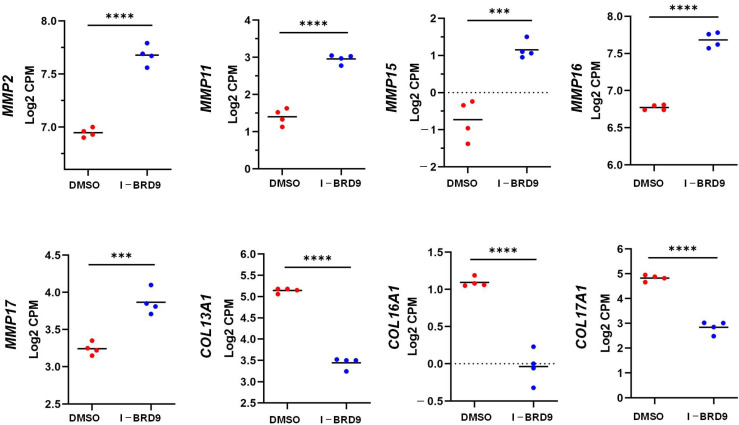
I-BRD9 altered the RNA expression of ECM-related genes in HuLM cells. RNA-seq revealed the upregulation of *MMP2*, *MMP11*, *MMP15*, *MMP16*, and *MMP17* and downregulation of *COL13A1*, *COL16A1,* and *Col17A1* in HuLM cells treated with I-BRD9. *** *p* < 0.001; **** *p* < 0.0001.

Several studies have demonstrated that abnormal ECM accumulation and remodeling are critical for UFs [42,43,44,45]. Excessive ECM deposition and production can contribute to mechanotransduction, therefore regulating downstream signaling leading to the pathogenesis of UFs [46,47,48]. With reactome pathway analysis, we demonstrated that I-BRD9 could activate matrix metalloproteinases, degrade the ECM, and alter the ECM organization, as shown in Figure 5. RNA-seq analysis revealed that gene expression levels of members of the metzincin superfamily—matrix metalloproteinases, including *MMP2*, *MMP11*, *MMP15*, *MMP16*, *MMP17* and *MMP24*—were upregulated in response to I-BRD9 treatment. In contrast, the expression levels of genes encoding collagen type XIII alpha 1 chain (*COL13A1*), collagen type XVI alpha 1 chain (*COL16A1*) and collagen type XVII alpha 1 chain (*COL17A1*) were downregulated via targeted inhibition of BRD9 (Figure 5). We validated the expression of several ECM-related genes via qPCR. The results are consistent with RNA-seq data, as shown in Figure S3B.

#### 2.4.3. Inhibition of BRD9 Altered the Gene Expression Correlating to Epigenetic Modifications

**Figure 6:**
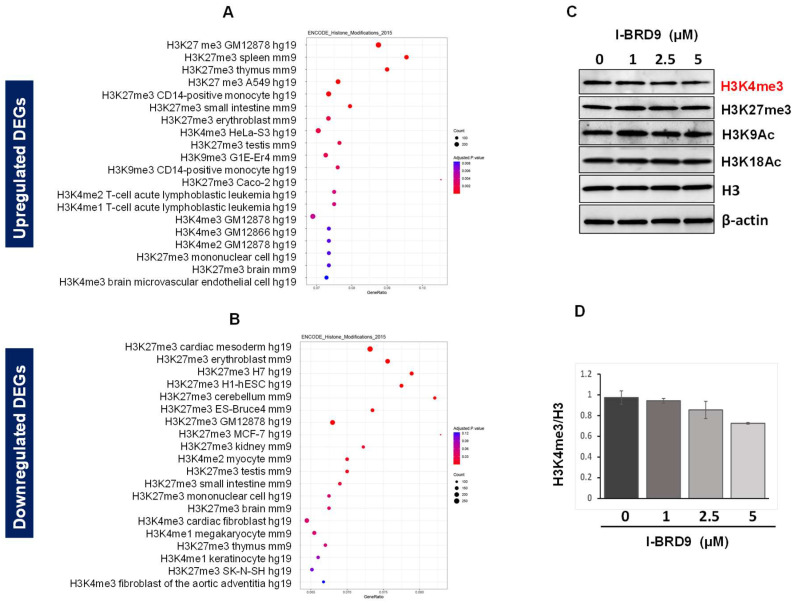
Enrichment analysis for histone modifications. The dot plots showed the top twenty enrichment terms for histone modification associated with up DEGs (**A**) and down DEGs (**B**) in response to I-BRD9 treatment. The *x*-axis represents the gene ratio, and the *y*-axis describes the enrichment components. The area of the circle is proportional to the number of genes assigned to the term, and the color accords with the adjusted *p*-value. (**C**) The levels of histone marks, including H3K4me3, H3K27me3, H3K9Ac, and H3K18Ac, were examined via immunoblot analysis in HuLM cells in the presence (I-BRD9 (1–5 µM) or absence (DMSO) of I-BRD9. (**D**) The levels of H3K4me3 were quantified in HuLM cells in the presence or absence of I-BRD9 using NIH Image J.

To investigate the relation between I-BRD9-induced DEGs and epigenome alterations in UF cells, we performed an enrichment analysis of epigenetic histone markers using the Enrichr web server. As shown in Figure 6, up DEGs between control and I-BRD9-treated HuLM cells were associated with H4K27me3 and H3K9me3, among others. Genes repressed via I-BRD9 were correlated with H3K27me3 and H3K4me3 (Figure 6A,B). To determine if the BRD9 inhibition directly alters the levels of histone marks, we performed Western blot analysis and compared the levels of four histone marks between control and I-BRD9-treated HuLM cells. As shown in Figure 6C, I-BRD9 treatment did not markedly alter the levels of histone marks, including H3K27me3, H3K9Ac, and H3K18Ac, except for H3K4me3. I-BRD9 at 5 µM decreased the H3K4me3 levels to 27.5% compared to the control (Figure 6D), indicating that I-BRD-9-induced DEGs were associated with specific histone mark installation. These studies suggest that inhibition of BRD9 may reshape the UF transcriptome to a favorable state.

**Figure 7:**
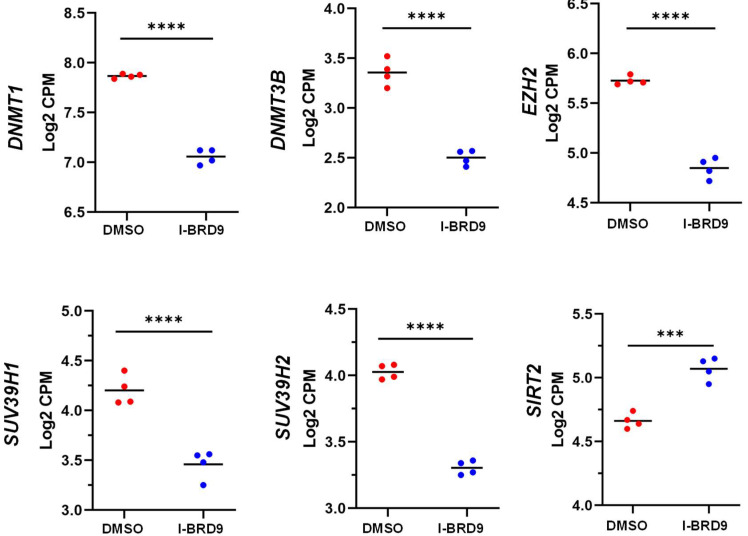
I-BRD9 altered the RNA expression of epigenetic regulators in HuLM cells. RNA-seq revealed the downregulation of *EZH2, SUV39H1*, *SUV39H2*, *DNMT3B*, *and DNMT1 and upregulation of SIRT2* in HuLM cells treated with I-BRD9. *** *p* < 0.001; **** *p* < 0.0001.

We performed targeted gene analysis using our RNA-seq data and found that the RNA expression of several epigenetic genes was altered in response to I-BRD9 treatment. These altered genes included *EZH2*, *SUV39H1*, *SUV39 H2*, *DNMT1*, *DMMT3B*, and *SIRT2* (Figure 7). We also validated the expression of several epigenetic genes via q-PCR, and the results are consistent with RNA-seq data (Figure S3C). These analyses suggest that I-BRD9 treatment may alter the transcriptome via multiple epigenetic mechanisms. 

#### 2.4.4. Inhibition of BRD9 Altered the Levels of m^6^A Regulators in UF Cells

**Figure 8:**
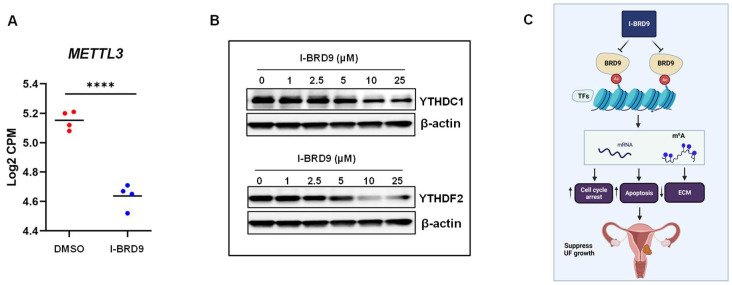
The expression of m^6^A writers in HuLM cells in response to I-BRD9 treatments and the experimental model. The RNA expression of *METTL3* (**A**) in HuLM cells treated with I-BRD9. (**B**) The comparison of protein levels of m^6^A readers (YTHDC1 and YTHDF2) in vehicle- and I-BRD9-treated HuLM cells. (**C**) The experimental model shows that I-BRD9 treatment promotes apoptosis, induces cell cycle arrest, represses ECM accumulation, and reprograms the epigenome and epitranscriptome in UF cells. Figure 8C was created using BioRender software (BioRender.com, accessed on 23 December 2023). **** *p* < 0.0001.

RNA modification impacts gene expression, and N6-methyladenosine (m^6^A) is the most pervasive, abundant, and conserved internal modification within eukaryotic mRNAs [49,50,51,52,53]. Accordingly, we examined the RNA expression of key m^6^A writer METTL3 in UF cells in the presence or absence of I-BRD9. As shown in Figure 8A, I-BRD9 treatment significantly decreased the RNA levels of METTL3 by RNA-seq analysis. In addition, we validated the expression of *METTL3* and *METTL14* genes via qPCR. The qPCR results for *METTL3* were consistent with RNA-seq data, as shown in Figure S3D. However, the expression of *METTL14* exhibited no significant difference between DMSO- and I-BRD9-treated HuLM cells. 

To further determine the connection between BRD9 and m^6^A regulators, we performed Western blot analysis to measure the protein levels of m^6^A readers in HuLM cells in the presence or absence of I-BRD9. As shown in Figure 8B, targeted inhibition of BRD9 decreased the protein levels of YTHDC1 and YTHDF2 dose-dependently.

## 3. Discussion 

Epigenetic alterations regulate gene activity and expression beyond the underlying DNA sequence and are linked to many diseases, including uterine tumorigenesis [6,54,55,56,57,58]. Therefore, understanding the relationship between epigenetic regulators and tumorigenesis is crucial for manipulating chromatin regulation in tumor therapy [59,60,61,62]. The present study revealed that BRD9, one of the key readers of lysine acetylation for regulating protein–histone association and chromatin remodeling, is aberrantly upregulated in UF tissues and cell lines. Furthermore, targeted inhibition of BRD9 induced cell cycle arrest and apoptosis, altered several critical biological pathways, including ECM accumulation, reprogrammed the pathological epigenome/epitranscriptome, and modulated gene regulation in UF cells. Notably, our data discriminated between cell proliferation of UF and myometrial cells in response to BRD9 inhibitor treatment. 

Prior functional and correlational studies on BRDs in tumor biology have been extensively investigated in several types of cancer [63]. One of the most significant advances in understanding the role of BRDs in human diseases focuses on the BET proteins, including BRD2, BRD3, BRD4, and BRDT, which share a common domain consisting of two N-terminal bromodomains, which bind to acetylated lysine residues on histones [64,65,66]. BRD2 is essential for proinflammatory cytokine production in macrophages [67]. BRD2 and BRD4 physically associate with the promoters of inflammatory cytokine genes in macrophages. JQ1, an inhibitor of the BET family of BRD proteins, including BRD2/4, can block this association and reduce IL-6 and TNF-a levels. These studies suggest that targeting the BET proteins could benefit hyperinflammatory conditions associated with high levels of cytokine production [67]. In ovarian cancer, JQ1 suppresses tumor growth associated with cell cycle arrest, apoptosis induction, and metabolic alterations [68]. In Ewing sarcoma, co-immunoprecipitation revealed an interaction of BRD4 with CDK9. Combined treatment of Ewing sarcoma with BRD- and CDK9-inhibitors resulted in enhanced responses compared to individual drugs in vitro and in a preclinical mouse model in vivo [69]. 

BRDs include BET and non-BET BRD families. In addition to the BET family, the role of the non-BET family members has been investigated recently. For instance, the non-BET family inhibitor NVS-CECR2-1 inhibits chromatin binding of CECR2 BRD and displaces CECR2 from chromatin within cells. NVS-CECR2-1 exhibits cytotoxic activity against various human cancer cells, killing SW48 colon cancer cells by inducing apoptosis [37]. In renal clear cell carcinoma, the combined analysis of BRD9 and other chromatin-regulated genes showed a significant association with the high-risk groups and lower overall survival, providing a prediction model for further research investigating the role of the expression of BRD genes in cancers [70]. Recently, the critical role of BRD9 in the pathogenesis of uterine leiomyosarcoma has been identified [36].

In contrast, the role and mechanism underlying the relevance and involvement of BRD family members in the pathogenesis of UFs, which are the most common benign reproductive tumors, are entirely lacking. Therefore, we characterized the functional role of BRD9, which may play an important role in UF progression. We investigated this specific protein initially because it was recently identified and involved in multiple diseases [71,72,73]. Notably, BRD9 has been shown to play an important role in the uterine cancer leiomyosarcoma [36]. Our studies demonstrated that BRD9 is aberrantly upregulated in UF tissues and cells, indicating that BRD9 may contribute to the pathogenesis of UFs. 

Epigenetic-targeted therapy has been applied in the treatment of various cancers [74,75,76]. Therefore, the development and use of small chemical inhibitors are fundamental and critical to the preclinical evaluation of BRD proteins as targets. In addition to BET protein inhibitors, recently, several inhibitors targeting BRD9 have been developed with high potency for BRD9 [30,77]. Notably, the BRD9-selective antagonist I-BRD9 has been employed in several types of cancer, including rhabdoid tumors [78], ovarian cancer [79], clear cell renal cell carcinoma (ccRCCs) [80], acute myeloid leukemia [81], and colon adenocarcinoma [72]. I-BRD9 demonstrated beneficial effects on tumor cell growth inhibition, and the combination of I-BRD9 with cytotoxic drugs resulted in additive to synergistic inhibitory effects. In addition, I-BRD9 has been tested in vivo [72,79,80], and single I-BRD9 administration effectively inhibited ccRCCs and colon cancer cell growth in tumor-bearing mice. Moreover, the I-BRD9 has tolerable in vivo toxicity profiles from these studies [41]. To determine the functional role of BRD9 in UFs, we investigated the effect of BRD9 potent inhibitor (I-BRD9) on UF cells. We demonstrated that I-BRD9 treatment significantly inhibited UF cell proliferation accompanied by increased cell cycle arrest and decreased ECM accumulation. In addition, the morphological changes with increased apoptotic cells were observed in response to I-BRD9 treatment, encouraging further investigation of I-BRD9’s effect in vivo. These observations are consistent with the previous studies on BRD9 inhibition from other types of cells [41,81] and contribute to our understanding of the molecular mechanism underpinning ECM deposition and cell proliferation mediated by BRD9’s involvement in UFs. 

To extensively determine the mechanism of BRD9 inhibitory action, we performed comparative transcriptome-wide RNA-sequencing in UF cells treated with vehicle or BRD9 inhibitor (I-BRD9). Our transcriptomic profiling analysis in UF cells revealed that multiple important pathways were altered in response to I-BRD9 treatment. For instance, I-BRD9 altered pathways in UF cells, including E2F targets, G_2_M checkpoint, MYC targets, MTORC1 signaling, and mitotic spindle and DNA replication. We have identified several cell cycle- and apoptosis-related genes that may play an important role in the inhibitory effect on UF cell growth, supporting the molecular mechanism underlying the BRD9 inhibition-induced anti-UF cell effect. We demonstrated that cyclin-dependent kinases and their associated pathways are impacted in response to BRD9 inhibition. Reactome pathway analysis revealed that BRD9 inhibition altered the ECM accumulation and remodeling, which are the key factors for mechanotransduction and downstream signaling alterations for UF pathogenesis and growth. Previously, transcriptome-wide mRNA profiling in melanoma cells demonstrated that BRD9 inhibition upregulated pro-apoptotic genes associated with the p53 pathway and downregulated several extracellular matrix proteins required for tumor growth [41,72,78,79,80,81].

It has been widely accepted that different epigenetic mechanisms coordinately regulate gene expression and function [82,83,84,85]. To determine the relation between BRD9 and histone marks, we analyzed the epigenomic alterations associated with DEGs upon I-BRD9 treatment. Notably, we observed that DEGs in response to I-BRD9 treatments were significantly related to the enrichment of several histone marks, including H3K27me3, H3K4me1, H3K4me2, and H3K4me3. In addition, BRD9 inhibition altered the expression of several epigenetic marks, including *EZH2*, *SUV39H1*, *SUV39H2, and SIRT2*. BRD proteins regulate gene expression through multiple mechanisms, including chromatin remodeling, histone modification, histone recognition, and transcriptional machinery regulation [23]. Notably, members of the BET protein family, including BRD2, BRD3, BRD4, and BRDT, have been reported to play a role in installing histone methylation. For instance, blocking the readers of H3K27ac via BET inhibitor (JQ1) abolished H3K27ac-induced H3K4me3 installation and downstream gene activation [86]. In addition to BET BRDs, our findings herein reinforce the view that non-BET BRDs, such as BRD9, may also play a critical role in cross-talking with histone modification ‘modifiers’ via the enrichment analysis of epigenetic modification. We observed that I-BRD9-induced DEGs are associated with epigenetic marks, such as H3K4me3 and H3K27me3. Using immunoblot analysis, we observed the marked protein level decrease in the detected histone mark, H3K4me3, suggesting that BRD9 inhibition may alter the installation of histone marks and that BRD9 is essential for global H3K4me3 maintenance. Importantly, abnormal H3K4me3 modifications revealed dysregulated Wnt/β-catenin and TGF-β pathways, ultimately promoting UF progression [10]. In addition, integrating ChIP-seq and RNA-seq data demonstrated that several proto-oncogenes and tumor suppressor genes were identified among the hypertrimethylated/up DEGs and the hypotrimethylated/down DEGs, respectively [10]. Therefore, our study suggested that the I-BRD9-induced inhibitory effect on UF cell proliferation may be attributed to reprogrammed vital pathways and genes via certain histone modifications. Notably, we also demonstrated that BRD9 inhibition can alter the expression of other epigenetic regulators, such as *DNMT1, DNMT3B, EZH2, SUV39H1, SUV39H2, SIRT2*, among others, indicating that BRD9 may functionally link to the epigenetic network. Notably, I-BRD9 treatment did not alter the H3K27me3 levels in HuLM cells, suggesting that BRD9 inhibition may alter H3K27me3-regulated genes via histone recognition or other mechanisms. Further studies are needed to determine the additional histone marks that may link to the BRD protein function. The detailed molecular/epigenetic mechanisms underlying the transcriptional changes of ECM-, apoptosis- and cell cycle-related genes in response to BRD9 inhibition also need to be explored. 

The epitranscriptome is an emerging frontier in molecular medicine owing to its vast potential as an additional and highly dynamic layer of gene regulation above and beyond the epigenome [87,88,89]. To date, more than 160 different chemical modifications in RNA have been identified in living organisms; among these, m^6^A is the most pervasive, abundant, and conserved internal modification within eukaryotic mRNAs, occurring in ~25% of transcripts genome-wide and enriched around stop codons, 5′- and 3′- untranslated regions, and within long internal exons. m^6^A is incorporated co-transcriptionally by so-called ‘writers’, including the METTL3-METTL14 core methyltransferase complex and associated proteins that confer target mRNA specificity, removed by the demethylases (erasers), and recognized by readers including the YTH family of proteins such as YTHDC1, and 2 [90,91,92,93]. m^6^A-bound readers ultimately alter the posttranscriptional fate of methylated mRNAs through modulation of cellular activities that control RNA stability, processing, and translation. m^6^A is thus a pervasive regulator of gene expression as well as a critical determinant of cell fate and function; accordingly, disruption of m^6^A homeostasis has been implicated in a growing number of pathological conditions, including cancer [94,95]. Importantly, m^6^A and its corresponding readers, erasers, and writers are emerging drug targets in various disease settings [92], suggesting a vast but untapped potential therapeutic reserve in UFs. A recent study demonstrated a link between BRD9 and m^6^A eraser FTO in ccRCCs [80]. Moreover, mRNA m^6^A has been shown to modulate gene expression through transcriptional regulation via histone modification [96,97]. In this study, we revealed that I-BRD9 resulted in a decrease in H3K4me3 and m^6^A reader levels. However, the connection between H3K4me3 and m^6^A readers, including YTHDC1 and YTHDF2, has not been explored. It is worthwhile to characterize how histone modifications and epitranscriptomic regulators coordinate to alter the transcriptome in the context of abnormal BRD protein function, and. targeting BRD9 in UFs might alter the post-transcriptional fate of RNA via epitranscriptomic and histone modification mechanisms, therefore modulating the cell function.

Given the findings presented in this study, we propose a mechanistic model for targeted inhibition of BRD9 in UFs herein that: (1) BRD9 expression is aberrantly overexpressed in UFs, (2) targeted inhibition of BRD9 reverses the UF phenotype with a decrease in cell proliferation and modulated ECM deposition and remodeling, (3) I-BRD9 modulates several key pathways, and reprograms the pathological epigenome and epitranscriptome, potentially leading to a new strategy for generating effective, precise non-hormonal treatment for UFs (Figure 8C).

This study has several limitations. The measurement of BRD9 expression was conducted in relatively small samples. UF heterogeneity occurs at many levels, including etiology, clinical symptoms, and pathogenesis, significantly impacting research design and therapeutic decisions [45]. In this study, we observed the variation of BRD9 expression in UFs. The variation can be discriminated even between multiple UFs from the same uterus. Notably, we also observed differential expression of BRD9 in MM across patients. Given the fact that the MM from the UF-containing uterus (MyoF) is different from MM from the uterus without UFs (MyoN) [1,98,99], heterogeneous UFs can impact the MM characteristics via the paracrine effect and mechanotransduction mechanisms variously. Additionally, an increased sample size with information on *MED12* mutation may address if aberrant BRD9 expression links to *MED12* mutation or other characteristics of UF. Although in vitro treatment with I-BRD9 was performed in both UF and myometrial cell lines, the effect of BRD9 bromodomain inhibition on cellular functions in UF-derived primary cells has not been explored. Future studies are encouraged to further determine the epigenetic mechanisms underlying BRD9 inhibition-induced transcriptome changes. ChIP-seq and whole-transcriptome maps of the internal m^6^A methylated nucleotide, such as MeRIP-seq and m^6^A-SAC-seq [100,101,102,103], can be adopted to determine the relation between BRD protein function and epigenetic regulation. In addition, single-cell transcriptome profiles can distinguish between cell cycle states in tumor cells [104]. Therefore, single-cell RNA-seq can characterize cell cycle-specific transcriptional changes attributed to BRD9 inhibition. 

In conclusion, our study demonstrated for the first time that BRD9 protein is aberrantly upregulated in UFs. Furthermore, inhibition of BRD9 suppresses the UF cell phenotype via altering vital UF-related pathways and reprogramming the pathological epigenome/epitranscriptome. Therefore, targeted inhibition of BRDs in UFs may provide a promising and novel strategy for treating patients with this clinically significant disease.

## 4. Materials and Methods

### 4.1. Sample Collection and Experimental Design

The study was approved by the University of Chicago’s Institutional Review Board (IRB 20-1414). Fibroid tissues were consistently collected from peripheral parts of large intramural fibroid lesions (>5 cm in diameter) with care to avoid areas of apparent necrosis, bleeding, or degeneration. Myometrium tissues were collected at least 2 cm away from the closest fibroid lesion. Patients underwent the informed consent process, and documented informed consent forms were collected and stored. Only records indicating that the patient had not used any hormonal treatment for at least three months before the surgery date were included. These UFs have a white, pear-shaped appearance. 

The bioinformatics analysis overview is shown in Figure S1.

### 4.2. Cells and Reagents

The immortalized human UF cell line (HuLM) and immortalized human uterine smooth muscle (UTSM) cell line were generous gifts from Dr. Darlene Dixon. These immortalized cell lines are non-tumorigenic in nude mice and exhibit no phenotypic alteration from the parental cell types [105]. The cells were cultured and maintained in phenol red-free, 10% fetal bovine serum Dulbecco’s Modified Eagle Medium: Nutrient Mixture F-12. In addition, the BRD9 inhibitor, I-BRD9, was purchased from Selleck Chemical (Cat# S7835, Houston, TX, USA). The range of doses tested was 1–25 µM.

### 4.3. Protein Extraction and Western Blot Analysis

Cells were collected and lysed in RIPA lysis buffer with protease and phosphatase inhibitor cocktail (Thermo Scientific, Waltham, MA, USA). The protein lysates from UF and adjacent myometrial tissues were prepared as described previously [106]. The protein was quantified using the Bradford method (Bio-Rad Protein Assay kit, Hercules, CA, USA). The information about primary antibodies, including antibody dilution and source of antibodies, is listed in Table 1. The antigen–antibody complex was detected with Trident Femto Western HRP substrate (GeneTex, Irvine, CA, USA). Specific protein bands were visualized using the BIO-RADS IMAGING SYSTEM (Bio-Rad, Hercules, CA, USA). Band signals were quantified using the NIH ImageJ software (version 1.53t, U. S. National Institutes of Health, Bethesda, MD, USA).

### 4.4. Proliferation Assay

Cell proliferation was measured using a trypan blue exclusion assay. Cells were seeded into 12-well tissue culture plates and treated with BRD9 inhibitor (I-BRD9) at a dose range of 1–25 µM for 48 h. An equal amount of DMSO was used as vehicle control. After treatment, the cells were trypsinized and collected via centrifuge. The cells were resuspended in a serum-free medium. An equal volume of 0.4% trypan blue and cell suspension was mixed and applied to a hemacytometer for cell counting. Viable cells were unstained. This assay was performed three times in triplicate.

### 4.5. Measurement of Cell Cycle Phase Distribution

Cell cycle distribution was determined via flow cytometric analysis as described previously [107]. Briefly, UF cells were cultured in DMEM/F12 medium containing 5 µM of I-BRD9 for 24 h. Control cells were cultured in a medium containing an equal amount of DMSO. Cells were then washed with PBS, fixed in 70% ethanol, and hypotonically lysed in 1 mL of DNA staining solution [0.05 mg/mL PI (Sigma) and 0.1%Triton X-100]. Cells were acquired at 12 µL/min on a LSRFortessa (Special Order Research Product, BD Biosciences) running DIVA v.8.0.2. Propidium iodide was excited via a 50 mw 561 laser, and the signal was collected through a 585/15 bandpass filter. Cell cycle analysis was performed using the Watson model included within FlowJo v10.8.1.

### 4.6. Assessment of Apoptosis

UF cells were cultured in DMEM/F12 medium in the presence or absence of I-BRD9. The live, dead, and apoptotic cells were differentiated during flow cytometry with FITC Annexin V/Dead Cell Apoptosis Kit (Invitrogen, Cat#V13242, Carlsbad, CA, USA).

### 4.7. RNA Sequencing

To determine the mechanism underlying the inhibitory effect of BRD9 inhibition on the UFs, the UF HuLM cells were treated with BRD9 inhibitor I-BRD9 (5 µM, *n* = 4) and DMSO vehicle control (*n* = 4) for 48 h. RNA was isolated using Trizol. RNA quality and quantity were assessed using the Agilent bio-analyzer. Strand-specific RNA-SEQ libraries were prepared using a TruSEQ total RNA-SEQ library protocol (Illumina provided, San Diego, CA, USA). Library quality and quantity were assessed using the Agilent bio-analyzer, and libraries were sequenced using an Illumina NovaSEQ6000 (illumine provided reagents and protocols). 

### 4.8. Transcriptome Profiles Analysis

#### 4.8.1. Transcriptome Data Analysis

The classical alignment-based mapper STAR, version 2.6.1d (GitHub, Inc., San Francisco, CA, USA) (23) was used to map sequencing reads to a human reference transcriptome using GRCh38p13. The results of STAR mapping were quantified with Salmon, version 1.4.0. Then, Bioconductor (https://bioconductor.org/packages/release/bioc/html/tximport.html, accessed on 27 October 2021) was used to read Salmon outputs into the R environment. Annotation data from Gencode V34 was used to summarize data from transcript-level to gene-level. A variety of R packages was used for this analysis. All graphics and data wrangling were handled using the tidyverse suite of packages. All packages used are available from the Comprehensive R Archive Network (CRAN), Bioconductor.org, or Github.

#### 4.8.2. Differential Gene Expression Analysis

To identify the differentially expressed genes (DEGs) between treatment and control groups, we selected three different methods: DESeq2 [108], edgeR [109], and Limma + voom [110]. We used a cutoff of −1.5 > fold change > 1.5 and a *p*-value of 0.05 for these three methods. In addition, Benjamini and Hochberg’s (BH) method was performed to control the false discovery rate of all the genes with an adjusted *p*-value of less than 0.05.

#### 4.8.3. Pathway Analysis of DEGs

We used GSEA Desktop Application v4.3.2 for gene set enrichment analysis (GSEA). We chose Hallmark and C2 curated gene sets from MSigDB to compare the impaired pathways between I-BRD9 and DMSO. A total of 1000 permutations were performed using gene sets, and the pathways with an FDR-*p* value ≤ 0.05 were chosen as significantly enriched.

#### 4.8.4. Functional and Regulatory Enrichment Analysis

Comprehensive gene list enrichment analysis for regulation machinery was carried out using the EnrichR [111] package in R. We used ENCODE Histone Modifications 2015 for histone modification enrichment in EnrichR to determine the mechanisms underlying the regulation of DEGs. 

### 4.9. RNA Extraction and Quantitative Real-Time Polymerase Chain Reaction (qRT-PCR)

Total RNA was isolated using Trizol reagent (Invitrogen). The concentration of total RNA was determined using NanoDrop (Thermo Scientific, Waltham, MA, USA). One microgram of total RNA from each sample was reverse-transcribed to complementary DNA (cDNA) using the High-Capacity cDNA Transcription Kit (Thermo Scientific, Waltham, MA, USA).

Quantitative real-time PCR was performed to determine the mRNA expression of genes as described previously [112]. Primers were purchased from Integrated DNA Technologies (IDT, Coralville, IA, USA), with primer sequences shown in Table 2. An equal amount of cDNA from each sample was added to the master mix containing appropriate primer sets and SYBR green supermix (Bio-Rad) in a 20 µL reaction volume. All samples were analyzed in triplicate. Real-time PCR analyses were performed using Bio-Rad CFX96. Cycling conditions included denaturation at 95 °C for 2 min, followed by 40 cycles of 95 °C for 5 s and 60 °C for 30 s, then 65 °C for 5 s. The synthesis of a DNA product of the expected size was confirmed via melting curve analysis. 18S ribosomal RNA values (internal control) were used to normalize the expression data, and normalized values were used to create data graphs. Negative control was performed by running the reaction without cDNA.

### 4.10. Statistical Analysis 

All experiments were conducted with biological replicates. A comparison of 2 groups was carried out using Student’s *t*-test for parametric distribution and the Mann–Whitney test for nonparametric distribution. Comparison of multiple groups was carried out via analysis of variance (ANOVA) followed by a post-test using Tukey’s test for parametric distribution and the Kruskal–Wallis test followed by a post-test Dunns for nonparametric distribution, using GraphPad Prism 9 Software. Data are presented as mean ± standard error (SE). In figures, *, **, ***, and **** indicate, *p* < 0.05, <0.01, <0.001, and <0.0001, respectively.

## Figures and Tables

**Table 1:** Antibodies were used in the study.

Antibodies	Company	Catalog#	Source	Application	Dilution	Size (KDa)
BRD9	Cell signaling	58906	Rabbit	WB	1:1000	80
PCNA	GeneTex	GTX100539	Rabbit	WB	1:1000	29
FN	cell signaling	26836	Rabbit	WB	1:1000	300
YTHDC1	Abcam	ab122340	Rabbit	WB	1:1000	85
YTHDF2	Abcam	ab220163	Rabbit	WB	1:1000	62
H3K4me3	Active Motif	39160	Rabbit	WB	1:1000	17
H3K27me3	Active Motif	39157	Rabbit	WB	1:1000	17
H3K9Ac	Active Motif	39918	Rabbit	WB	1:1000	17
H3K18Ac	Active Motif	39756	Rabbit	WB	1:1000	17
H3	Cell Signaling	4499	Rabbit	WB	1:2000	17
β-actin	Sigma	A5316	Mouse	WB	1:5000	42

**Table 2:** Primers were used in the study.

Gene Symbol	Primer Sequences	F or R	Assay	Species	Size (bp)	Accession
*CDK2*	AGATGGACGGAGCTTGTTATC	F	q-PCR	Human	103	X62071
*CDK2*	CTTGGTCACATCCTGGAAGAA	R	q-PCR	Human	103	
*CCND1*	GGGTTGTGCTACAGATGATAGAG	F	q-PCR	Human	112	NM-053056.3
*CCND1*	AGACGCCTCCTTTGTGTTAAT	R	q-PCR	Human	112	
*CCND3*	GTGTTGTCCCTTCTAGGGTTATT	F	q-PCR	Human	102	M92287.1
*CCND3*	TGAGAGGAGCCATCTAGACTATT	R	q-PCR	Human	102	
*PCNA*	GGACACTGCTGGTGGTATTT	F	q-PCR	Human	105	J04718
*PCNA*	CAGAACTGGTGGAGGGTAAAC	R	q-PCR	Human	105	
*Col3A1*	CTGGGCTTCCTGGTTTACAT	F	q-PCR	Human	106	NM_001130103.2
*Col3A1*	GCTCCTTGGTCTCCCTTATC	R	q-PCR	Human	106	
*Col17A1*	TTGTCCGTAGGCCCATACTA	F	q-PCR	Human	113	NM_000494.4
*Col17A1*	CCTCTTCTCCCTTTATTCCTTCC	R	q-PCR	Human	113	
*MMP11*	TCCTGACTTCTTTGGCTGTG	F	q-PCR	Human	114	NM_005940.5
*MMP11*	CATGGGTCTCTAGCCTGATATTC	R	q-PCR	Human	114	
*MMP15*	CTGCTCCAGACAGGGAATTAG	F	q-PCR	Human	139	NM_002428.4
*MMP15*	CAAAGAGAGCCTGGCAGTTA	R	q-PCR	Human	139	
*MMP16*	GACATACATCCCAACCTCTCTC	F	q-PCR	Human	97	NM_005941.5
*MMP16*	ACAGGCAATACCCATCATACTC	R	q-PCR	Human	97	
*DNMT1*	CGGCCTCATCGAGAAGAATATC	F	q-PCR	Human	95	NM_001130823.3
*DNMT1*	TGCCATTAACACCACCTTCA	R	q-PCR	Human	95	
*DNMT3B*	GGAGCCACGACGTAACAAATA	F	q-PCR	Human	98	NM_006892.4
*DNMT3B*	GTAAACTCTAGGCATCCGTCATC	R	q-PCR	Human	98	
*SIRT2*	GGACAACAGAGAGGGAGAAAC	F	q-PCR	Human	120	AY030277.1
*SIRT2*	AGACAAGAACTGCTGGTTAAGA	R	q-PCR	Human	120	
*SUV39H1*	CGAGGAGCTCACCTTTGATTAC	F	q-PCR	Human	122	NM_001282166.2
*SUV39H1*	CAATACGGACCCGCTTCTTAG	R	q-PCR	Human	122	
*METTL3*	CACTGATGCTGTGTCCATCT	F	q-PCR	Human	131	NM_019852.5
*METTL3*	CTTGTAGGAGACCTCGCTTTAC	R	q-PCR	Human	131	
*METTL14*	CCTGGGAATGAAGTCAGGATAG	F	q-PCR	Human	119	NM_020961.4
*METTL14*	CCCAGGGTATGGAACGTAATAG	R	q-PCR	Human	119	
*18S*	CACGGACAGGATTGACAGATT	F	q-PCR	Human	119	NR_145820
*18S*	GCCAGAGTCTCGTTCGTTATC	R	q-PCR	Human	119	

## Data Availability

Raw FASTQ files were deposited in the NCBI Gene Expression Omnibus (GSE195800).

## Supplementary Materials

The following supporting information can be downloaded at: https://www.mdpi.com/article/10.3390/ijms25020905/s1.
