# Supplementary figures and images for: Bromodomain-Containing Protein 9 Regulates Signaling Pathways and Reprograms the Epigenome in Immortalized Human Uterine Fibroid Cells
Source: Int J Mol Sci. 2024 Jan 11;25(2):905. doi: 10.3390/ijms25020905 (PMC10815284; doi:10.3390/ijms25020905)

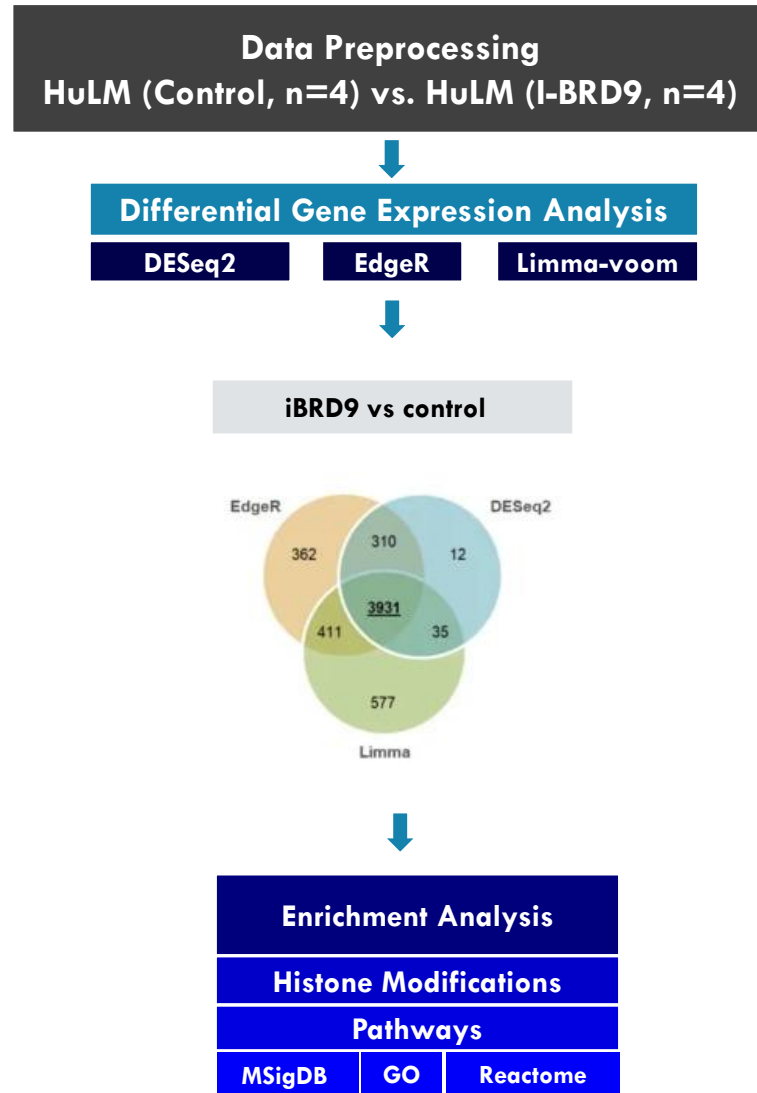

Fig. S1

**A**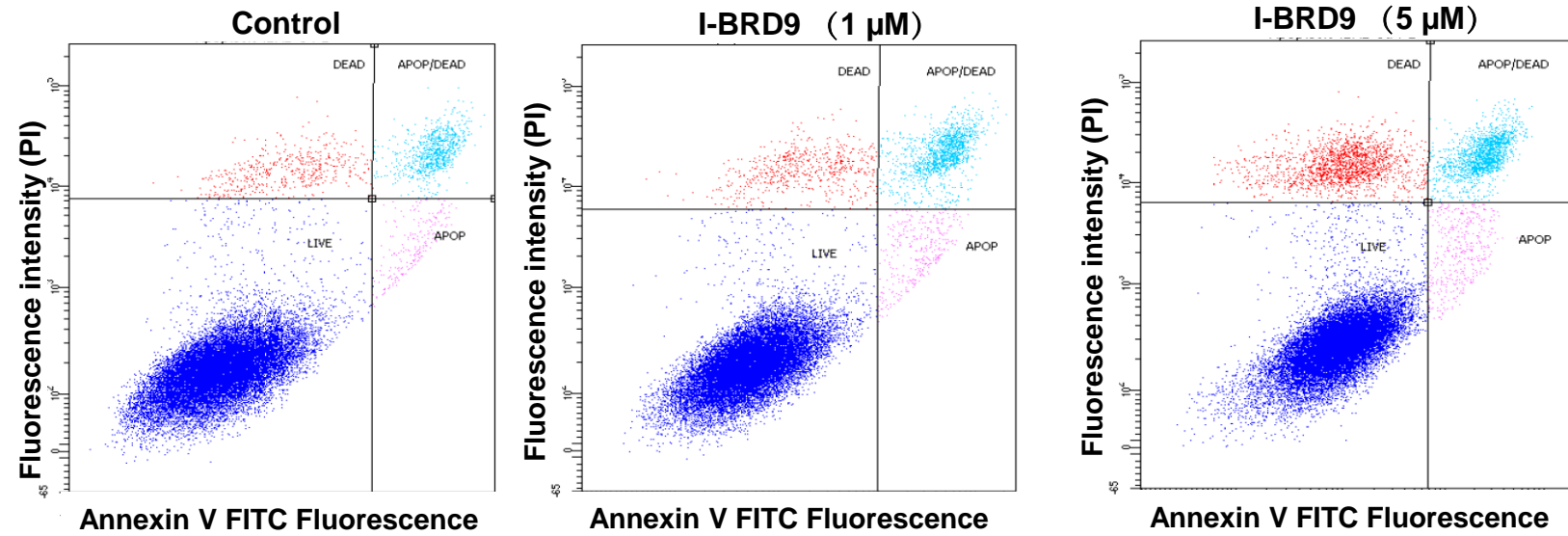**B**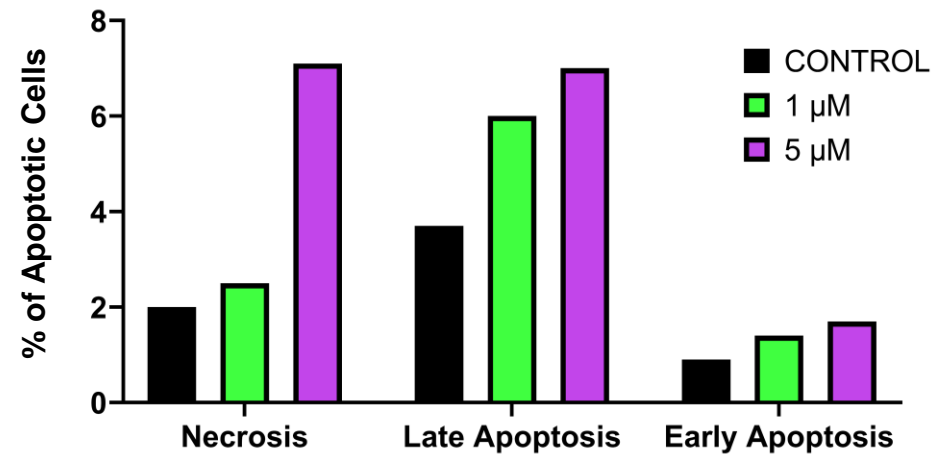

Fig. S2

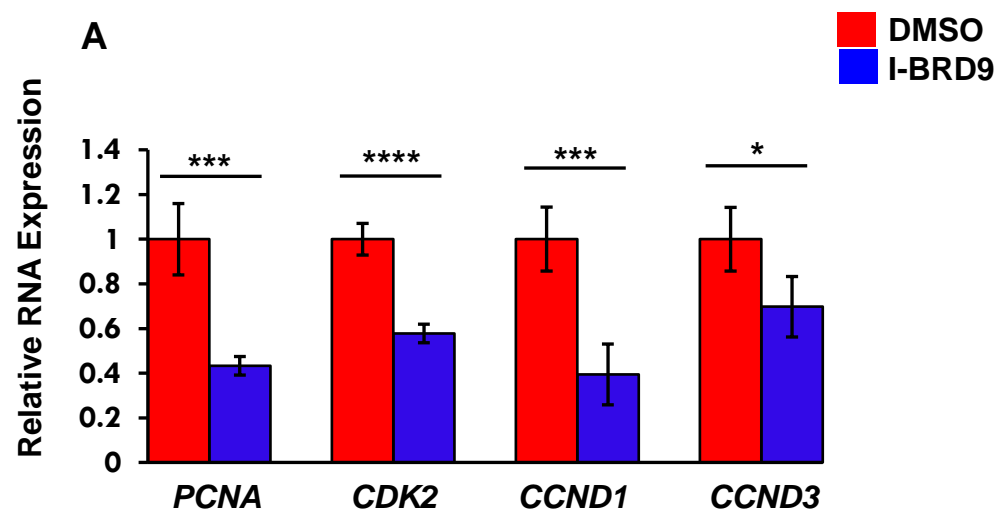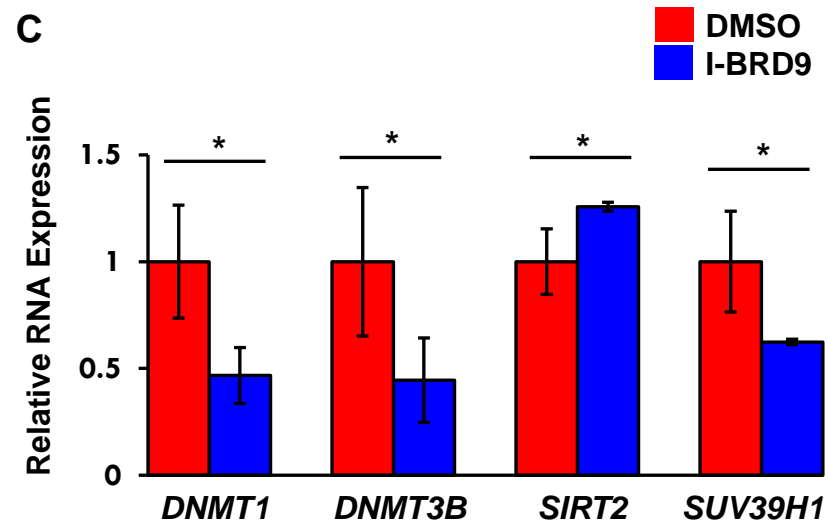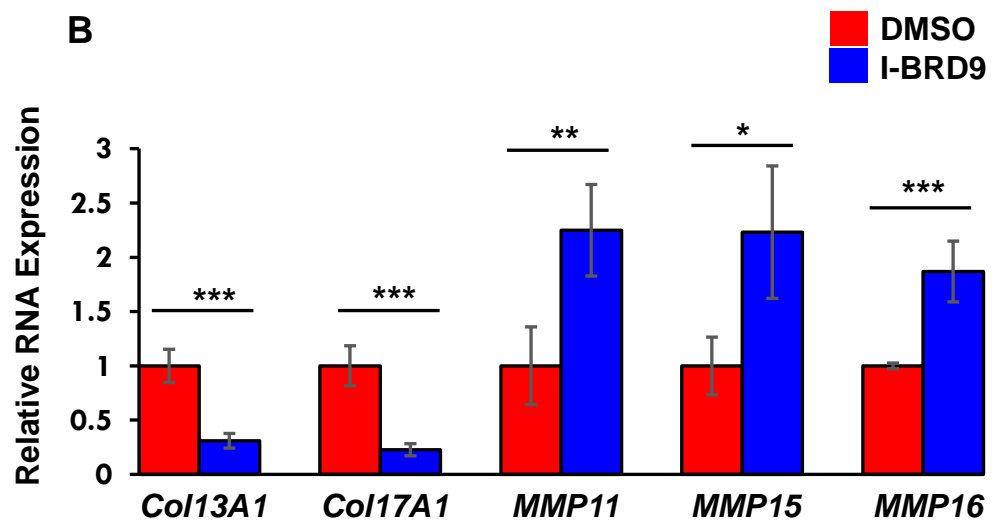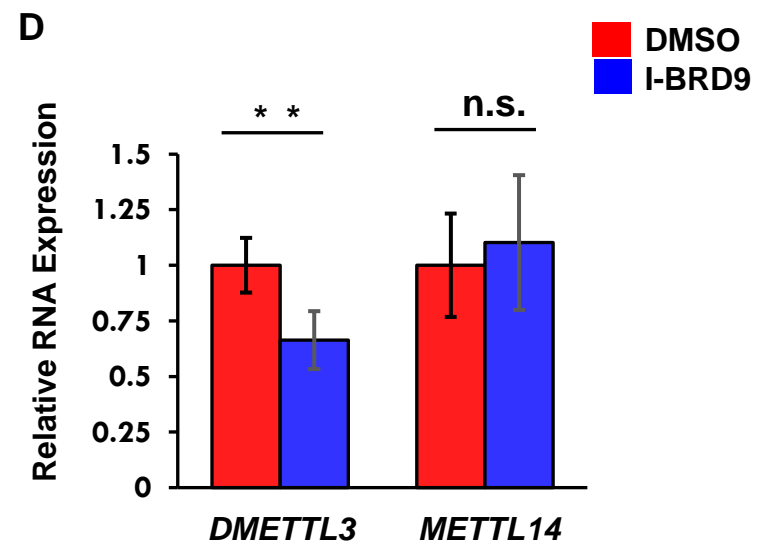

Fig.S3

Supplement: Supplementary file 1 [file ijms-25-00905-s001.zip › ijms-2685678-supplementary.pdf]
